# What Is the Relationship between Metacognition and Mental Effort in Executive Functions? The Contribution of Neurophysiology

**DOI:** 10.3390/bs13110918

**Published:** 2023-11-10

**Authors:** Michela Balconi, Carlotta Acconito, Roberta A. Allegretta, Davide Crivelli

**Affiliations:** 1 International Research Center for Cognitive Applied Neuroscience (IrcCAN), Faculty of Psychology, Università Cattolica del Sacro Cuore, 20123 Milan, Italy; michela.balconi@unicatt.it (M.B.); carlotta.acconito1@unicatt.it (C.A.); robertaantonia.allegretta1@unicatt.it (R.A.A.); 2 Research Unit in Affective and Social Neuroscience, Department of Psychology, Università Cattolica del Sacro Cuore, 20123 Milan, Italy

**Keywords:** metacognition, cognitive effort, executive functions, EEG, mental fatigue

## Abstract

Prolonged cognitive effort can be considered one of the core determinants of mental fatigue and may negatively affect the efficacy and efficiency of cognitive performance. Metacognition—understood as a multi-componential set of skills concerning awareness and control of one’s own cognition—might reduce such negative outcomes. This study aimed to explore the relation between metacognitive skills, neurocognitive performance, and the level of mental effort as mirrored by electrophysiological (EEG) markers of cognitive load and task demand. A challenging cognitive task was used to prompt and collect metacognition reports, performance data (accuracy and response times—RTs), and physiological markers of mental effort (task-related changes of spectral power for standard EEG frequency bands) via wearable EEG. Data analysis highlighted that different aspects of metacognitive skills are associated with performance as measured by, respectively, accuracy and RTs. Furthermore, specific aspects of metacognitive skills were found to be consistently correlated with EEG markers of cognitive effort, regardless of increasing task demands. Finally, behavioral metrics mirroring the efficiency of information processing were found to be associated with different EEG markers of cognitive effort depending on the low or high demand imposed by the task.

## 1. Introduction

Mental fatigue is a state characterized by a decline in cognitive performance and increased subjective feelings of exhaustion. It can result from prolonged periods of cognitive activity and is often associated with tasks that require sustained attention and effort. Mental effort is linked and follows the allocation of cognitive resources to complete a task, with the level of effort varying based on task demands (in turn modulated, for example, by task complexity, time pressure, ambiguity of stimuli and/or instructions), individual factors (e.g., subjective profile of neurocognitive efficiency, weariness, level of psychophysical stress or physical strain), and situational circumstances (e.g., physical characteristics of the environment as well as affective connotations of the situation in which the task has to be faced) [1,2,3,4,5,6].

Studies investigating neurofunctional correlates of executive control and strategic management of neurocognitive resources have shown that the dorsolateral prefrontal cortex (dlPFC) plays a central role in their allocation and in the regulation of mental effort [7]. Consistently, neuroimaging studies have shown that mental fatigue also is associated with changes in prefrontal activation patterns [8,9,10,11]. Initially, there is increased activation in regions responsible for attention and effort, such as the prefrontal cortex, anterior cingulate cortex, and parietal cortex, during mentally fatiguing tasks. Over time, during such tasks, these areas may show reduced activation, suggesting a neural basis for the decline in performance.

Cognitive strategies such as goal setting and time management techniques, besides higher cognitive functions such as strategic planning, situated reasoning, and decision-making, support and mediate the efficiency of mental operations needed to face and solve challenging tasks as expressions of an intentional and goal-directed regulation of mental effort [12].

Relevantly for this discussion, such manifestation of the self-regulation meta-function is a core constituent of metacognitive skills, contributing to cognitive achievement and performance notwithstanding increasing mental effort and fatigue via its association with motivational factors and processes [13,14,15]. In keeping with a multi-componential model of metacognitive skills, it is therefore possible to identify two core facets of metacognition: knowledge of cognition and regulation of cognition [16,17]. The first one—“knowledge of cognition”—refers to all that is knowable and referable. In other words, it concerns knowledge of one’s thoughts, capabilities, and strategies, as well as the appropriate time to use and modify them. On the other hand, the second facet—“regulation of cognition”—identifies with the capacity for monitoring and control of own thoughts, feelings, and behavior. Specifically, it includes higher cognitive functions such as planning, evaluating, and monitoring activities that influence cognitive processes, as well as flexibly adapting strategies in relation to the outcomes of different situations. This second facet could also be defined as a meta-level process in that the reflexive processes involved in observing and regulating cognitive processes are responsible for cognitive control, which is especially essential for effectively and efficiently completing various decision-making and higher-cognition tasks [18,19,20].

Regarding, the above-introduced second facet of metacognition, attention regulation can be considered a pervasive and overarching higher-order process that results from the interactions of complex dynamic modalities that are functionally connected by a single cognitive activity [21]. Therefore, from this viewpoint, it is possible to characterize the control of attention as a necessary skill for humans to be able to respond to novel and complex environmental demands. Being reflexively aware of the mental processes supporting attention control and orienting, as well as consistently exerting strategies to up-regulate and/or optimize those processes, represent critical advantages in effectively managing challenges imposed by everyday life, especially when the balance between task demands and individual resources is altered, and mental fatigue grows.

Given the relevance and potential for optimizing human performance, developing reliable methods to assess mental effort and metacognitive control skills, as well as understanding the relationship between those two constructs, represent highly valuable challenges. Therefore, several tasks based on shifting or maintaining focus as well as blocking automatic behaviors were developed to try and investigate an individual’s capacity for cognitive control and attention regulation [22]. Further, the neuroscientific perspective, which combines the study of the individual’s explicit and behavioral responses with the study of implicit brain and autonomic responses, has remarkably contributed to the investigation of those skills. EEG [23] and autonomic measure recordings [24] are just two examples of neuroscientific tools that enable the measurement of a person’s typical neuro- and psycho-physiological responses linked to executive control processes leading to effective attention regulation, highlighting levels of cognitive effort and emotional engagement [25]. EEG, specifically, allows for detecting the electrical activity of the brain and determining the potential cognitive load and mental effort imposed by a task, thanks to the functional significance of frequency bands. As an example, it was found that changes in high-alpha power mirror task difficulty, plausibly marking increased cortical activation due to a greater neural effort when higher task demands require further cortical processing [26,27,28]. Beta synchronization is a known marker of cortical activation aimed at managing task-related workload, endogenous top-down regulation of attention, and, generally, mental resources [29,30,31,32]. Again, modulation of theta power over frontal sites was associated with task demand, plausibly marking the recruitment of attentional resources for working memory, action monitoring, and focusing [26,33,34,35].

The increasing diffusion of wearable technologies is furthering applied research and will likely offer novel insights into understanding and mitigating mental fatigue and optimizing mental effort in various contexts. As an example, wearable EEG devices now allow for measuring brain electrical activity noninvasively and easily, providing real-time data on neural markers of mental fatigue and effort, such as on changes in brainwave patterns [36]. Yet, such opportunities have yet to be fully explored in order to, for example, investigate the relationship between cognitive performance, executive control processes linked to the management of mental effort, and metacognition—understood as awareness and regulation of the strategies employed to efficiently complete a cognitive task—in real-life contexts. Indeed, to the best of our knowledge, no previous studies have explored whether individual metacognitive skills, executive control performance, and physiological markers of mental effort are associated, and in such cases, to what extent.

To begin filling this gap, this study aimed to explore the relation between metacognitive skills, neurocognitive performance, and level of mental effort as mirrored by EEG markers of cognitive load and task demand, and to investigate whether greater metacognition is associated with better performance. To achieve this goal, a neuroscientific approach was adopted based not only on the administration of a revisited attentional switching task but also on the continuous detection of neurophysiological parameters via wearable EEG.

Based on the above-introduced background knowledge, we expected to observe a positive correlation between metacognitive skills and performance at the challenging task. Also, even though this investigation is still explorative given the lack of previous evidence, we expected to observe significant correlation coefficients even between metacognitive skills and EEG task-related markers of cognitive load, mirroring the pervasive modulatory role of metacognitive processes not only on behavior but also on neurofunctional correlates of performance. Finally, we expected to observe consistent profiles when looking at physiological and behavioral markers of performance and mental fatigue, with worse performance (e.g., increased RTs) associated with increased EEG markers of cognitive load (e.g., greater task-related beta band power).

## 2. Materials and Methods

### 2.1. Sample

This study involved a sample of 24 participants (Male = 13, Female = 11; M_age_ = 35.3, SD_age_ = 11.7). None of the recruited participants had neurocognitive impairments, a history of psychiatric or neurological illnesses, or were currently receiving pharmacological therapy that could alter neurofunctional responses or impair cognition or judgment. Additionally, participants reported normal or corrected-to-normal vision, normal global cognitive functioning, and no habitual drug taking. Also, participants were asked to refrain from smoking and drinking coffee in the two hours before testing, in order to limit potential effects on cognitive performance related to such habits (should they have them). Any of these requirements that failed to be satisfied led to exclusion from the study.

Finally, to take part in this study, participants signed a written informed consent and agreed to participate voluntarily without receiving any compensation. The Ethics Committee of the Department of Psychology at the Università Cattolica del Sacro Cuore approved the research protocol and its methods, which comply with the principles and regulations in the Helsinki Declaration (2013).

### 2.2. Procedure

The experiment took approximately 20 min and was conducted in a dedicated quiet room, where the participants were invited to sit in a comfortable chair in front of a monitor (distance: approximately 80 cm). After filling and signing the written informed consent, the experimental procedures and the task were presented to the participants. Specifically, before beginning the experimental task, participants were asked to wear a non-invasive EEG recording band, to detect neurofunctional activity at rest and during task performance.

#### 2.2.1. Behavioral Data Acquisition: Task and Data Processing

The experimental task was administered via a web-based experiment-management platform (PsyToolkit, version 3.4.4; [37]) and consisted of two different phases: the task performance phase and the metacognition assessment phase.

In the task performance phase, participants performed a digitalized challenging dual-task, in which they had to respond to two different tasks depending on whether stimuli were presented at the top or bottom of the screen. Specifically, if the stimulus appeared at the top of the screen the response had to be given based on its shape, whereas if it was presented at the bottom of the screen the response had to be given based on the number of dots depicted within the stimulus. In both situations, to correctly perform the task, it is necessary to ignore the feature of the stimulus (i.e., the shape or the number of dots) that is not relevant for such trial and orient cognitive resources to process the relevant one, depending on the part of the screen where the stimulus is presented. If the stimulus was presented at the top of the screen, thus making shape information relevant, participants had to discriminate between squared and diamond shapes by using two different buttons. When the stimulus appeared at the bottom of the screen, thus making information concerning the number of dots relevant, the same two buttons had to be used to discriminate between two- or three-dot patterns. During the task, participants were also asked to try and metacognitively focus on their performance and cognitive strategies (if any) used during the task.

**Figure 1:**
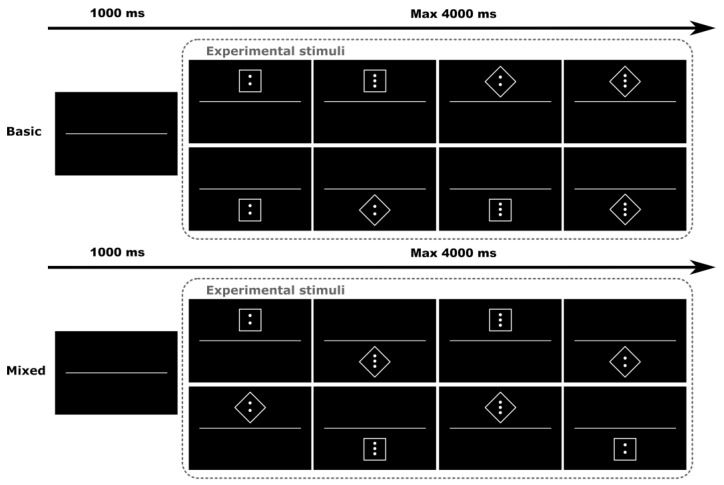
Trial structure for the computerized dual-task, basic, and mixed conditions.

The task included a total of 84 trials, in which the stimulus was presented until a response was given and up to a maximum duration equal to 4000 ms, with 1000 ms of interstimulus interval (see Figure 1). Also, the task included two blocked sequential conditions: basic and mixed. In the basic condition block (number of trials = 42), participants were instructed to pay attention to the top (or bottom) of the screen and had to perform one task at a time. In the mixed condition block (number of trials = 42), stimuli randomly appeared at the top and bottom of the screen and participants had to manage the demands of a dual-task and respond accordingly. For both conditions, mean accuracy and RTs were collected as behavioral metrics of performance, respectively mirroring the efficacy in responding to the task and the efficiency of related information processing.

The metacognition assessment phase consisted of the administration of a post-experiment quantitative questionnaire devised to assess metacognition and, specifically, participants’ awareness, use, and evaluation of strategies employed to complete the challenging digitalized task (adapted from: [38]). The metacognitive questionnaire included five items investigating: (i) the development and implementation of a strategy while performing the task (question: “*Were you able to apply some kind of strategy during the game?*”); (ii) the awareness of employing a strategy while performing the task (question: “*Were you aware of using this strategy during the game?*”); (iii) the occurrence of online self-monitoring and change in strategy while performing the task (question: “*Did you change your strategy during the game?*”); (iv) the perception of the effectiveness of the adopted strategy (question: “*Do you think you used an effective strategy?*”); and (v) the evaluation of the used strategy and of the opportunity to modify it (question: “*Would you make changes to the strategy used?*”). For each item, participants provided a response on a 5-point Likert scale, where 1 corresponds to “totally disagree” and 5 to “totally agree”.

#### 2.2.2. EEG Data Acquisition: Tool and Data Processing

**Figure 2:**
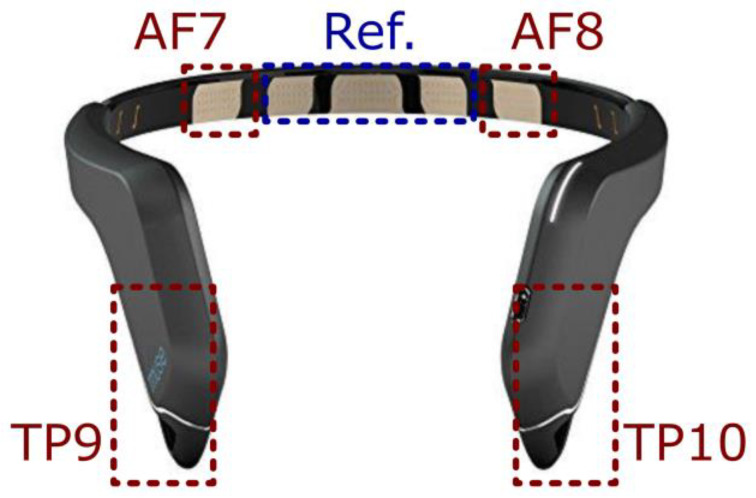
Wearable EEG device and related dry electrodes montage. Note. Ref.: Reference electrodes.

To collect EEG data non-invasively and to preserve the ecological validity of the study, the Muse^TM^ headband (version 2; InteraXon Inc., Toronto, ON, Canada) was employed in the resting-state recording and during the task to monitor and collect variations in EEG spectral activity (standard-frequency band power: delta, theta, alpha, beta, and gamma). This wearable EEG band included seven dry electrodes, three of which were used as references while the remaining four electrodes were used to collect the EEG signal from frontal (AF7 and AF8, respectively, left and right) and temporoparietal (TP9 and TP10, respectively, left and right) sites (see Figure 2).

Using the mobile app Mind Monitor, data were gathered and transferred via Bluetooth to an associated smartphone. The sampling rate was set to 256 Hz, and an online 50 Hz notch filter was applied during recording. Raw data were converted in real time in Power Spectral Density (PSD) via Fast Fourier Transformation, then mean PSD values were computed for each standard frequency band: delta (1–4 Hz), theta (4–8 Hz), alpha (8–13 Hz), beta (13–30 Hz), and gamma (30–44 Hz). Then, the average PSD for each frequency band was extracted for both baseline (eye-open and eyes-closed resting, duration for each condition: 120 s) and task-related conditions (basic and mixed conditions, duration for each condition: approximately 120 s on average). Finally, task-related changes in power spectral values were computed separately for the basic and mixed conditions, as follows:

TR_PSD_ = ((PSD_task_ − PSD_open-bl_)/PSD_open-bl_)

where TR_PSD_ represents task-related changes in power spectral values for each frequency band and each electrode site, PSD_task_ represents power spectral values in basic or mixed conditions for each frequency band and each electrode site, and PSD_open-bl_ represents power spectral values in eye-open baseline for each frequency band and each electrode site.

### 2.3. Data Analyses

The relations between metacognitive skills as measured via the items of the metacognitive questionnaire, neurocognitive performance in basic and mixed conditions as measured via accuracy and RTs metrics, and level of mental effort in basic and mixed conditions as measured via EEG markers of cognitive load and task demand (task-related changes of power spectral values for EEG bands) were explored by computing Pearson correlation coefficients.

Data analyses in this study were planned according to two sequential steps: the first one was focused on metacognition, while the second one was focused on performance. Specifically, in the first step, participants’ responses to the items of the metacognitive questionnaire were correlated with behavioral measures of neurocognitive performance (accuracy and RTs in basic and mixed conditions) and task-related changes of power spectral values for EEG bands (electrodes: AF7, AF8, TP9, TP10; EEG bands: delta, theta, alpha, beta, and gamma, condition: basic and mixed). In the second step, Pearson correlation coefficients were computed between performance metrics (accuracy and RTs) in each condition of the task (basic and mixed) and task-related changes of power spectral values for EEG bands (electrodes: AF7, AF8, TP9, TP10; EEG bands: delta, theta, alpha, beta, and gamma; condition: basic and mixed). The outcomes of correlation analyses were checked against the false discovery rate by applying the Benjamini–Hochberg procedure [39], so to minimize the risk of interpreting results biased by potential multiple testing errors.

## 3. Results

### 3.1. Step One: Metacognition

**Figure 3:**
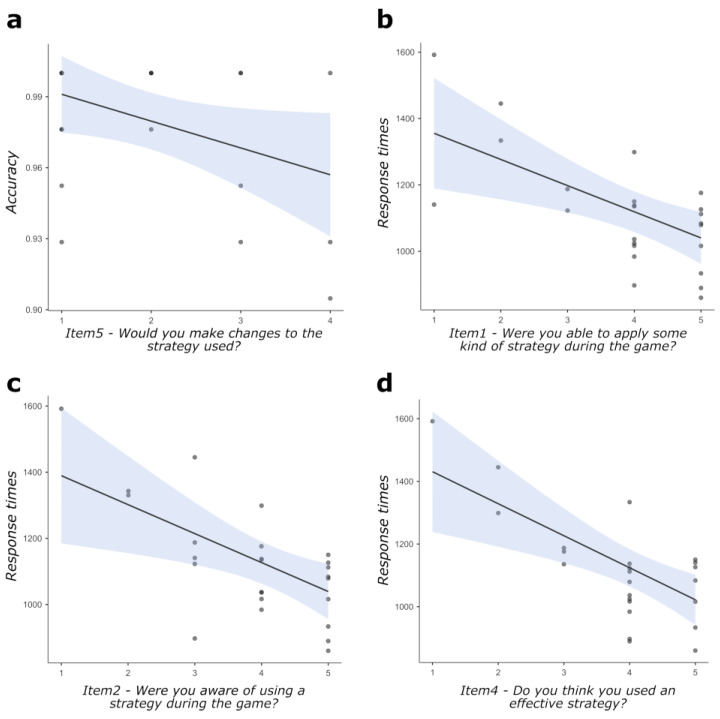
Scatterplots for statistically significant correlations between: (**a**) scores at metacognitive item 5 and accuracy in the mixed condition; (**b**) scores at metacognitive item 1 and RTs in the mixed condition; (**c**) scores at metacognitive item 2 and RTs in the mixed condition; and (**d**) scores at metacognitive item 4 and RTs in the mixed condition. The straight lines represent the global linear trends, while the shades represent their 95% confidence intervals.

Correlation analyses computed on the items of the metacognitive questionnaire and performance indices (accuracy and RTs) for basic and mixed conditions of the task highlighted: a significant negative correlation, between scores at item 5 and accuracy in the mixed condition (r = −0.405, *p* = 0.050; Figure 3a); a significant negative correlation between scores at item 1 and RTs in the mixed condition (r = −0.565, *p* = 0.004, Figure 3b); a significant negative correlation between scores at item 2 and RTs in the mixed condition (r = −0.527, *p* = 0.008; Figure 3c); and a significant negative correlation between scores at item 4 and RTs in the mixed condition (r = −0.605, *p* = 0.002; Figure 3d). No other correlation coefficient was found to reach the statistical threshold.

**Figure 4:**
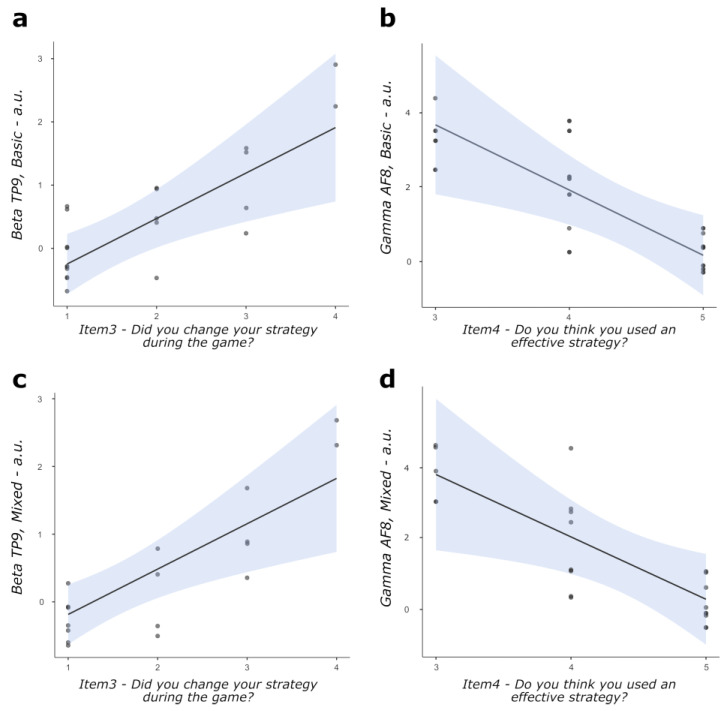
Scatterplots for statistically significant correlations between: (**a**) scores at metacognitive item 3 and task-related modulation of beta power at TP9 site in the basic condition; (**b**) scores at metacognitive item 4 and task-related modulation of gamma power at AF8 site in the basic condition; (**c**) scores at metacognitive item 3 and task-related modulation of beta power at TP9 site in the mixed condition; and (**d**) scores at metacognitive item 4 and task-related modulation of gamma power at AF8 site in the mixed condition. The straight lines represent the global linear trends, while the shades represent their 95% confidence intervals.

Correlation analyses computed on scores at metacognition items and EEG data for basic and mixed conditions of the task highlighted: a positive significant correlation between scores at item 3 and beta TRPSD for the basic condition in TP9 (r = 0.732, *p* = 0.004, Figure 4a); a negative significant correlation between scores at item 4 and gamma TRPSD for the basic condition in AF8 (r = −0.764, *p* = 0.010, Figure 4b); a positive significant correlation between scores at item 3 and beta TRPSD for the mixed condition in TP9 (r = 0.733, *p* = 0.004, Figure 4c); and a negative significant correlation between scores at item 4 and gamma TRPSD for the mixed condition in AF8 (r = −0.683, *p* = 0.020, Figure 4d). No other correlation coefficient was found to reach the statistical threshold.

### 3.2. Step Two: Performance

Correlation analyses computed between performance indices (accuracy and RTs) and EEG data for basic and mixed conditions of the task highlighted: a negative significant correlation between RTs and theta TR_PSD_ in AF8 for the basic condition (r = −0.616, *p* = 0.014); and a positive significant correlation between RTs and beta TR_PSD_ in AF8 for the mixed condition (r = 0.707, *p* = 0.010). No other correlation coefficient was found to reach the statistical threshold.

## 4. Discussion

The rationale of the present study was built on the potential of metacognitive skills and strategic regulation of mental effort for preserving performance in case of increasingly challenging conditions. Also, given the lack of systematic investigation of the relationship between metacognition and behavioral/neurophysiological markers of mental effort, we aimed to explore the relation between individual propensity towards metacognitive awareness and control, neurocognitive performance, and neurofunctional correlates of cognitive load and effort. Specifically, a challenging digitalized dual-task was used to prompt and collect metacognition reports concerning performance at the task and strategies used to complete the task, behavioral performance data, and electrophysiological markers of mental effort via wearable EEG.

Analysis of data highlighted three main findings: (i) metacognitive reports on the post-performance evaluation of the used strategy and of the opportunity to modify it, on intentional implementation and awareness of used task-solving strategies, and on their perceived efficacy correlated with performance as measured by accuracy and RTs; (ii) specific aspects of metacognitive skills we explored, such as online self-monitoring and adaptation of task-solving strategies and perception of the effectiveness of the adopted strategy, consistently correlated with EEG markers of cognitive effort (beta and gamma power modulations) as measured throughout the dual-task; and (iii) RTs correlated with different EEG markers of cognitive effort—namely, task-related changes in theta and beta power over frontal areas—depending on the low vs. high demand imposed by the task.

As for the first main finding, data suggest that gradually increasing individual propensity towards *reviewing* their own performance after its completion and towards *changing the used task-solving strategy* was linked to limited efficacy in responding to the task, as mirrored by lower accuracy scores. At the same time, individual propensity towards actually *using a task-solving strategy*, *online awareness* of implementing it, and *evaluation of efficacy* for the implemented strategy were linked to greater efficiency of information processing in responding to task demands, as mirrored by lower RTs. Such observations, taken together, seem consistent with a twofold organization of metacognitive skills paralleling the distinction between self-regulation and self-awareness metacognitive dimensions, in keeping with multicomponential accounts of metacognition (see, as examples, [16,17,18,19]).

As for the second main finding, correlations between individual propensity towards online monitoring and adaptation of implemented strategies to solve the task and individual perception of the effectiveness of such strategies, on the one side, and objective markers of cognitive load and effort during the task, on the other side, were observed. Relevantly, such correlations could be observed throughout the task in both the basic and the most cognitively demanding conditions.

More specifically, a greater propensity towards online monitoring and adaptation of implemented strategies to solve the task proved to be associated with greater task-related indices of beta activity over left temporoparietal sites. Beta oscillations are known markers of cognitive workload, endogenous attention focuses on a task, and top-down regulation of mental resources [29,30,31,32]. Furthermore, the power of the EEG beta band over left posterior sites was found to positively correlate with perceived mental effort during challenging cognitive tasks [40]. Notably, it was also suggested that such increased beta band power could mirror increased arousal levels needed to maintain attention during the tasks, in line with clinical observations of decreased beta band power and increased levels of effort in individuals presenting impairments of self-regulation and management of arousal (e.g., people showing attention deficit hyperactivity disorder, [40,41]). Building on such premises, we suggest that greater posterior beta activity related to the task, mirroring attention-related processes to increase arousal and orient cognitive resources on the task, might have marked individual performances connoted by the need for thorough monitoring and by online changes of the strategy used to respond to task demands.

At the same time, individual performances connoted by greater perceived effectiveness of implemented strategies were associated with lower task-related indices of gamma activity over right frontal sites. Frontal gamma activity was interpreted as an index of the dynamic between mental fatigue and sustained attention [42], vigilance [43], and greater cognitive performance [44]. Relevant to the present discussion, frontal gamma power has also recently been linked to the activity of a multilevel attention control system sensitive to changes in both low-level (such as visual cues) and high-level (such as task-related strategy) cognitive processes [45], in keeping with the cognitive-energetical framework focusing on the effects of stress and high workload on human performance [46], with the known relationship between frontal areas and attention processes [43,47], and with the known informativity of EEG in studying top-down and bottom-up control of mental resources [48,49]. In line with these recent theorizations, we propose that lower frontal gamma activity associated with higher perceived efficacy in the challenging task might mirror a reduced exploitation of neurocognitive resources to regulate attention during the task, which might have acted as an implicit cue for explicit reflective assessment of effectiveness in completing the task.

Finally, as for the third main finding, an analysis focusing on performance and investigating the relationship between behavioral and electrophysiological markers of mental effort pointed out a different pattern of correlations depending on the demand imposed by the two task conditions. Namely, RTs proved to be negatively correlated with task-related changes in theta power for the basic condition and to be positively correlated with task-related changes in beta power for the most demanding mixed condition. In both cases, relevant EEG modulations were located in the right frontal areas.

Modulations of theta power over frontal sites were associated with cognitive control of performance [50,51,52] and with the recruitment of attentional resources for working memory, action monitoring, and focusing to face task demands [26,33,34,35]. Furthermore, increased midfrontal theta coherence has been linked to lower variability of RTs [53], suggesting better monitoring and control over responses. Taken together, such evidence supports the interpretation of the inverse relation between theta activity and RTs in light of the positive effect of cognitive control over the efficiency of information processing. In other words, greater cognitive control, as marked by higher frontal theta activity, was coherently associated with better cognitive performance at the basic level of the task. Conversely, in the more complex condition of the task, performance as measured via RTs showed a significant correlation with beta activity. As noted above, EEG oscillations in the beta frequency range are deemed to mirror cortical activation aimed at managing task-related workload and top-down regulation of mental resources [29,30,31,32]. In keeping with such established evidence base, the direct relation between beta activity over frontal areas and RTs may plausibly be interpreted as a consistent manifestation of increased mental effort, signaled at both the behavioral (increasing effort as marked by greater RTs) and neurofunctional (increasing effort as marked by greater task-related beta activity) levels. Nonetheless, we acknowledge that the lack of significant correlations between theta activity and performance even as the level of challenge increases remains an open question. Future investigations could make this peculiar and differential pattern of low- vs. high-frequency pairings with performance their main focus of analysis, in order to try and parse out whether it could be traced back to different cognitive control mechanisms adaptively activated based on task demands.

Additionally, among the study limitations, we acknowledge that data interpretation and robustness of conclusions would benefit from further research with larger cohorts and replications. Furthermore, future studies could also take into account the subjective perception of cognitive effort as an additional source of information, as well as individual personality traits (e.g., self-consciousness, self-awareness, openness to experience) or cognitive styles (e.g., field dependence/independence, reflective-impulsive style, need for cognition) as potential mediators of the relationship between metacognitive skills, performance, and mental effort. Again, further depth into the discussion could be reached by integrating data on task-related metacognitive skills with qualitative reports on the actual strategy that participants have planned to use or used to solve the task. This could enrich data interpretation and give the opportunity to begin exploring even the potential role of logical/practical soundness of such strategy in influencing performance, awareness, and regulation. Finally, future applied studies should test the efficacy of empowerment protocols targeting metacognitive skills in improving the effective management of mental effort and fatigue.

## 5. Conclusions

This work is, to the best of our knowledge, among the first ones to focus on the relationship between metacognitive skills, performance, executive control of cognitive resources, and task-related neurofunctional markers of neurocognitive effort. Practical implications of a deeper understanding of the relationship between metacognitive awareness and regulation of performance, efficacy and efficiency of performance, and mental effort—as well as of the potentially protective role of metacognitive skills against cognitive fatigue—hint at the potential of the study.

## Data Availability

Data available from the authors upon reasonable request.
